# Assessing the agreement of chronic lung disease of prematurity diagnosis between radiologists and clinical criteria

**DOI:** 10.1186/s40748-024-00178-4

**Published:** 2024-04-05

**Authors:** Joseph Matthew Rich, Lydia Jing Lin, Jonathan Luan Le, Justin Ryan Ching Abe, Amit Sura

**Affiliations:** 1 USC-Caltech MD/PhD Program, Keck School of Medicine, University of Southern California, 1975 Zonal Ave, 90033 Los Angeles, CA USA; 2 Keck School of Medicine, University of Southern California, Los Angeles, CA USA; 3 John A. Burns School of Medicine, University of Hawaii, Honolulu, HI USA; 4 Department of Radiology, Children’s Hospital Los Angeles, Los Angeles, CA USA

**Keywords:** Bronchopulmonary dysplasia, Chronic lung disease of prematurity, Chest x-ray, Definitions, Infant, Premature

## Abstract

**Background:**

Chronic lung disease of prematurity (CLD) is the most prevalent complication of preterm birth and indicates an increased likelihood of long-term pulmonary complications. The accurate diagnosis of this condition is critical for long-term health management. Numerous definitions define CLD with different clinical parameters and radiology findings, making diagnosis of the disease ambiguous and potentially inaccurate.

**Methods:**

95 patients were identified for this study, as determined by the diagnosis or confirmation of CLD in the impression of the radiologist’s report on chest x-ray. Pulmonary function and complications were recorded at multiple benchmark timeframes within each patient’s first few months of life and used for determining eligibility under each definition.

**Results:**

Each clinical definition of CLD had a high sensitivity for patients identified to have CLD by radiologists, correctly fitting over 90% of patients. Most patients included required invasive mechanical ventilation or positive pressure ventilation at 36 weeks postmenstrual age, indicating patients with radiographically confirmed CLD tended to have more severe disease. Radiologists tended to diagnose CLD before 36 weeks postmenstrual age, a timepoint used by multiple standard clinical definitions, with cases called earlier fitting under a larger percentage of definitions than those called later.

**Conclusions:**

Radiologists tend to diagnose CLD in young patients with severe respiratory compromise, and can accurately diagnose the condition before developmental milestones for clinical definitions are met.

**Supplementary Information:**

The online version contains supplementary material available at 10.1186/s40748-024-00178-4.

## Introduction

**Fig. 1:**
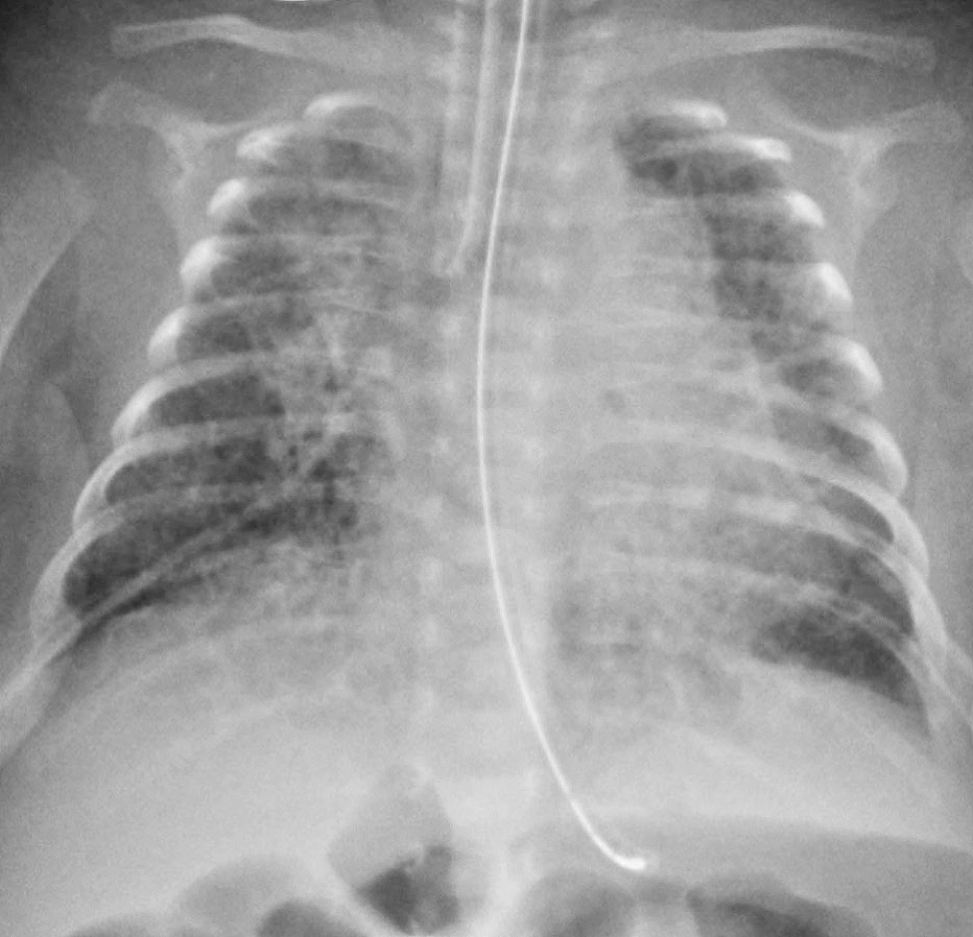
35 post-menstrual week old female with chronic lung disease. Single front view of the chest shows multifocal atelectasis superimposed on increased interstitial opacities with superimposed cystic lucencies consistent with chronic lung disease

Chronic lung disease of prematurity (CLD), formerly known as bronchopulmonary dysplasia, is the most prevalent complication of prematurity, affecting approximately 10,000–15,000 infants in the US each year [1]. Extremely premature infants are especially susceptible to CLD, with an incidence of 43% for preterm infants 28 weeks gestational age or less [2]. The etiology of CLD is due to prolonged mechanical ventilation and oxygen therapy for neonatal respiratory distress syndrome, causing inflammation of lung tissue [3]. The clinical presentation of CLD typically includes breathing difficulties and oxygen desaturation episodes within the first few months of life, and chest x-ray (CXR) classically displays fine granular or interstitial opacities [4] (Fig. 1). The condition implicates long-term health complications and impacts lifelong health management [5–7], necessitating the accurate diagnosis of the condition.

The disease was first characterized in 1967 with severe, acute respiratory distress and a high death rate [8]. Over time, new phenotypes of CLD have emerged with a range of severities due to both increased acuity of diagnosis and improved management and treatment of neonatal complications [9]. Numerous definitions have emerged in an attempt to define CLD objectively [10, 11]. One of the earliest definitions involved the presence of both radiologic abnormalities and difficulty with gas exchange at 30 days postnatal age [12]. In 1988, Shennan et al. defined CLD simply as the need for supplemental oxygen at 36 weeks postmenstrual age (PMA) [13]. The National Institute of Child Health and Human Development (NICHD) held a workshop in 2001 to modify Shennan’s definition, adding the need for at least 28 cumulative days of supplemental oxygen and defining a range of severity based on the degree of oxygen use at 36 weeks PMA [14]. Further studies added physiologic pulmonary tests to these diagnostic criteria [15, 16]. Isayama et al. extended Shennan’s definition in 2017 to needing supplemental oxygen at 40 weeks PMA [17]. The NICHD met in 2018 to modify their 2001 definition, removing the 28 cumulative days of oxygen requirement and adding a more complex scaled definition based on exact oxygen requirements and radiographic confirmation of parenchymal lung disease [18]. Jensen et al. added a simplified scale to Shennan’s definition in 2019 based on specific oxygen requirements at 36 weeks PMA (degree of nasal cannula or invasive mechanical ventilation) [19]. There is wide variation in these definitions, making objective and consistent diagnosis of the condition complex [10].

Multiple studies have compared some of these standard definitions of CLD and their ability to predict outcomes [10]. Saengrat et al. compared the NICHD 2018 to NICHD 2001 definition, finding the 2018 definition to have lower sensitivity for diagnosis but a better ability to predict mortality and pulmonary morbidities [20]. Katz et al. found similar abilities of the NICHD 2001, NICHD 2018, and Jensen definitions to predict neurodevelopmental and respiratory outcomes [21]. Pérez-Tarazona et al. found that the NICHD 2018 and Jensen definitions were better able to predict respiratory outcomes than the NICHD 2001 and Isayama definitions, and that the prevalence of CLD ranged from 49 to 70% among the four definitions [22]. Li and Xu found that the Jensen definition could better predict health expenditures than the NICHD 2018 definition [23]. With several definitions to compare and multiple possible metrics of success, it is challenging to find a single unified definition that should be applied.

The variation in these definitions has led to CLD being broadly applied by physicians whenever a preterm infant presents with pulmonary complications and radiologic signs of parenchymal lung disease, potentially leading to overuse of the diagnosis. To the best of our knowledge, this is the first time anyone has focused specifically on the diagnostic decision-making of radiologists in determining CLD on CXR analysis. The goal of this study was to determine how the diagnoses of radiologists compare with numerous standard definitions of CLD, providing insight into the concordance of the definitions based on radiologic analysis and determining if there is a need for a change in the diagnostic decision-making process.

## Materials and methods

We reviewed the charts of pediatric patients at Children’s Hospital Los Angeles (CHLA) between January 2010-May 2023 in whom the term “chronic lung disease of prematurity” or “bronchopulmonary dysplasia” appeared in the impression of the radiologist’s report of their chest x-ray. We used the database Montage, a radiology database hosted by CHLA, for searching through radiographs. We consider a radiologist to be making or confirming a diagnosis of CLD when this term appears in the findings and/or impression of the radiology report. Our exact search term was as follows:

(@impression(“bronchopulmonary dysplasia”| (“chronic lung disease of prematurity”| (“chronic lung disease” & (premature| prematurity| preterm)))))

105 patients were found with these criteria. Generally, in these cases, the radiologist made the diagnosis of CLD independently based on patient age (> 28 days), history of prematurity (< 31 weeks PMA at birth), and imaging findings (increased interstitial opacities with superimposed cystic lucencies); however, sometimes neonatologists were involved through the indication of CLD in the patient’s pertinent history. We reviewed the PowerChart electronic medical records of the eligible patients, and recorded key information necessary in determining eligibility under the various definitions of CLD (Supplementary Table 1). Data collected included gestational age and postmenstrual age at birth, oxygen requirements at 28 days postnatal, cumulative days of oxygen, oxygen requirements at 36 weeks PMA, and oxygen requirements at 40 weeks PMA. Additionally, we noted the date of diagnosis of the condition by the radiologist, which we defined as the earliest day in which the term “chronic lung disease” or “bronchopulmonary dysplasia” appeared in the impression of the report of the chest x-ray without already being included in the patient’s background. 10 patients were excluded for not having recorded information in the relevant timeframe (e.g., from outside hospital transfers), leaving us with 95 eligible patients for our study. We picked the following eight definitions to analyze based on prevalence in the community and commonality in the literature: Shennan (oxygen use at 36 weeks PMA), NICHD 2001 (at least 28 cumulative days of oxygen, with grading based on oxygen or respiratory requirements at 36 weeks PMA), Isayama (oxygen or respiratory requirements at 40 weeks PMA), NICHD 2018 (a graded definition which considers the degree of oxygen or respiratory requirements at 40 weeks PMA, radiologic confirmation, and parenchymal lung disease), Jensen (oxygen use at 36 weeks PMA with grading), oxygen use at 28 days postnatal, respiratory support at 36 weeks PMA, and oxygen use or respiratory support at 36 weeks postnatal. The satisfaction of each definition was automatically calculated based on the entered clinical data.

Statistical significance was determined by Chi-square testing and one-way analysis of variance (ANOVA) for categorical and discrete data, respectively. Chi-squared testing was used to determine the presence of significant differences in agreement among definitions as well as between pairs of definitions. One-way ANOVA was used to compare the average number of definitions fulfilled by multiple groups and was followed up by multiple comparison tests to determine which groups were significantly different. Statistical metrics and comparisons were computed in statistical analysis software (GraphPad Prism version 10.0.0 for Mac, GraphPad Software, Boston, Massachusetts, USA).

The full data table, including clinical data, radiologic information, and definition, can be found in Supplementary Table 1.

## Results

Patient demographics are described in Table 1. The majority of the patient population is male and born significantly preterm, with an average gestational age of 26 weeks. Gestational age ranged from 22 to 39 weeks, although only two patients were born at term past 37 weeks. The majority were still alive at the time of the study, and of those who passed, the vast majority (89%) were a result of respiratory causes. Almost all patients required some form of oxygen therapy at 36 weeks PMA, with the majority being on invasive mechanical ventilation. Most pertinent patient data was readily available, with the lowest reporting rate being for oxygen at 36 weeks PMA and 40 weeks PMA. As a result, the definitions which relied heavily on these parameters, including Shennan, NICHD 2001, and Isayama, had the lowest rates of accessibility, ranging between 80 and 93% (Supplementary Fig. 1).

**Table 1:** Patient demographics

Sex (% male):	71.58%
birthweight (kg)	0.88
gestational age (PMA at birth in weeks)	26.33
Length of hospital stay (weeks)	28.08
Recorded early respiratory death	16.84%
Age of diagnosing scan (weeks)	7.89
Invasive mechanical ventilation at 36 weeks PMA	67.37%
Noninvasive continuous PPV at 36 weeks PMA	15.79%
NC at 36 weeks PMA	8.42%

**Fig. 2:**
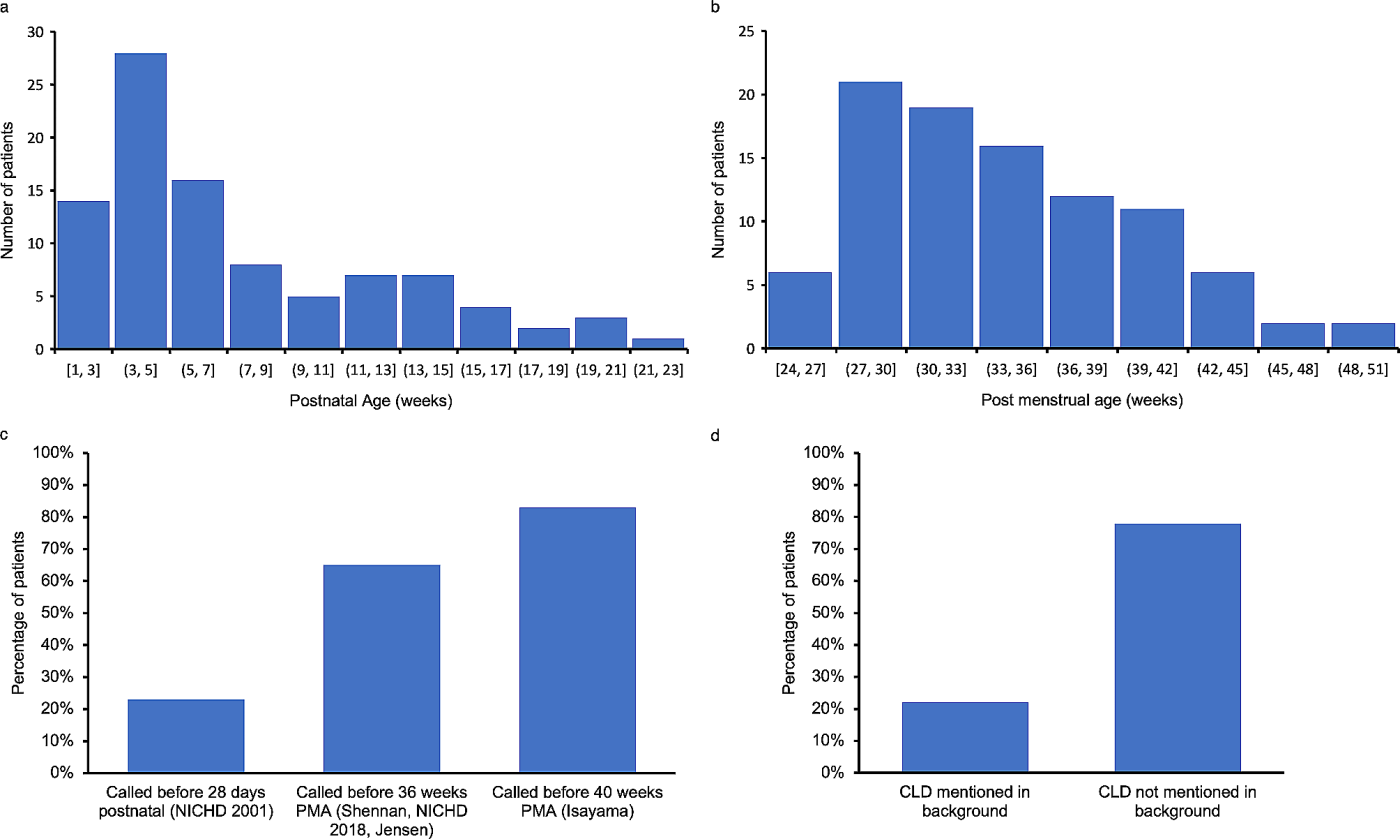
Radiologic diagnosis information. a Postnatal age at the time of radiologic diagnosis. b Postmenstrual age at the time of radiologic diagnosis. c Percentage of cases diagnosed after each of the major timeline milestones. d Percentage of cases which already included chronic lung disease in the patient background

The average postnatal age of patients at the time of radiologic diagnosis was 7.9 weeks, with a mode of approximately three to five weeks and a right-skewed distribution (Fig. 2a). The average PMA of patients at the time of radiologic diagnosis was 34.2 weeks, with a mode of approximately 27 to 30 weeks and a right-skewed distribution (Fig. 2b). Relative to the three major time point milestones for the CLD definitions (28 postnatal gestational days, 36 weeks PMA, 40 weeks PMA), many cases were diagnosed before these time points were reached. 23% of cases were called before 28 days postnatal, 65% were diagnosed before 36 weeks PMA, and 83% were diagnosed before 40 weeks PMA (Fig. 2c). Some common descriptors of findings consistent with chronic lung disease in the findings and impression of radiology report include “diffuse interstitial opacities” and “bronchovascular markings.” CLD was only previously mentioned in the background of the report in 22% of cases (Fig. 2d).

**Fig. 3:**
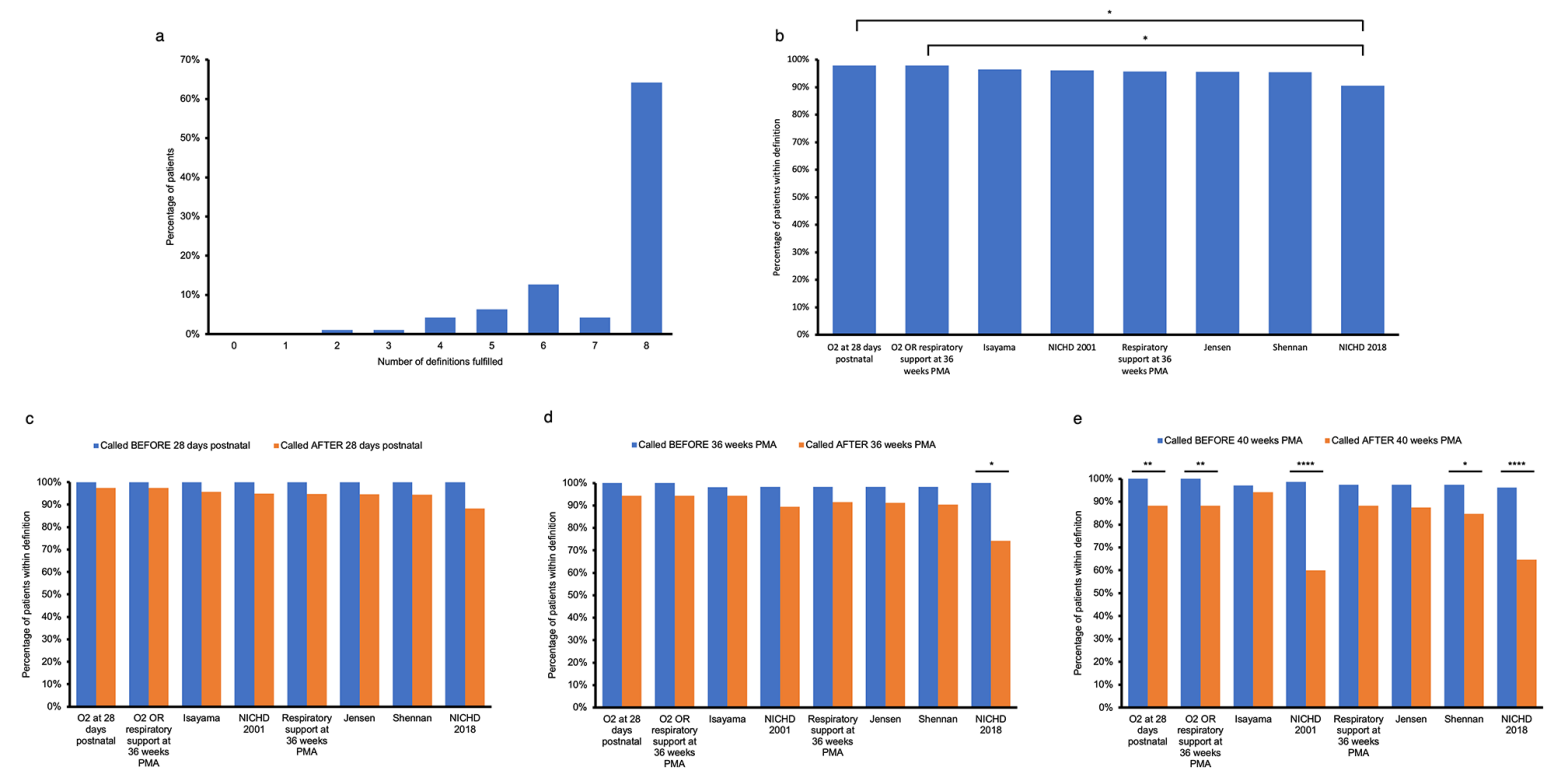
Correctness per definition. a Distribution of the percentage of definitions fulfilled per patient. b Percentage of patient population which fits into each definition. c Percentage of patient population which fits into each definition, stratified by whether the diagnosis was made before or after 28 gestational days. d Percentage of patient population which fits into each definition, stratified by whether the diagnosis was made before or after 36 postmenstrual weeks. e Percentage of patient population which fits into each definition, stratified by whether the diagnosis was made before or after 40 postmenstrual weeks

**Fig. 4:**
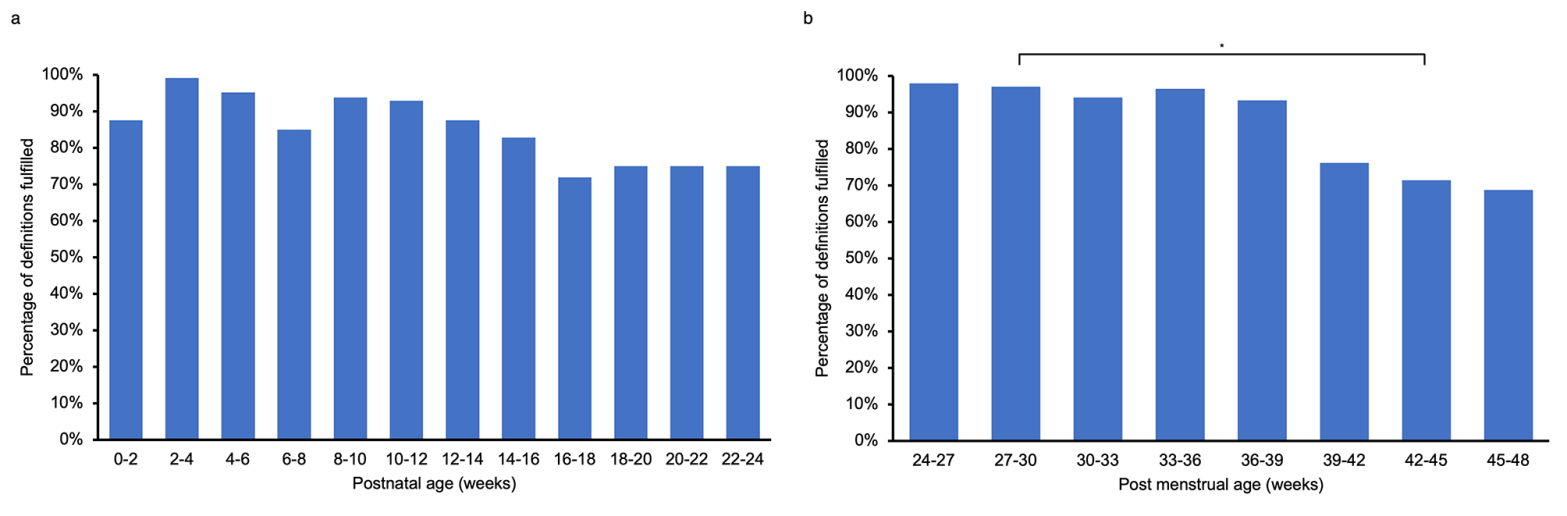
Radiology diagnostic accuracy by patient age at the time of diagnosis. a Diagnostic accuracy by postnatal age. b Diagnostic accuracy by postmenstrual age

Generally, these patients diagnosed with CLD by radiologists fit well into the clinical definitions. 64% of patients were classified as having CLD under all eight conditions, and 79% were classified as having CLD under six or more definitions (Fig. 3a). All definitions correctly captured the CLD diagnosis in at least 90% of patients, indicating high sensitivity of diagnosis from radiology (Fig. 3b). While overall, there was no significant difference across all definitions in the percentage of patients satisfying definition criteria (X2 = 8.491, *p* = 0.2913), the most sensitive definitions were those which checked oxygen requirements at 28 days gestational age or oxygen requirements or respiratory support at 36 weeks PMA. The least sensitive definition was NICHD 2018, which is the only definition with a statistically significant difference in diagnosis rate compared to the most sensitive definitions (X2 = 4.652, *p* = 0.0310) (Fig. 3b). Each pair of definitions was assessed for their level of agreement, defined as the percentage of cases where both definitions reach the same diagnosis (i.e., CLD or no CLD) out of the 95 total cases. When comparing the degree of agreement between each pair of definitions, the lowest rates of agreement would generally arise due to a combination of lower individual definition sensitivity and different evaluation criteria (Table 2). When comparing the sensitivity of diagnosis when called before vs. after the required time point, we find that cases diagnosed before the necessary time point actually had equal or better predictive abilities than those diagnosed after (Fig. 3c-e). For instance, NICHD 2018 had better predictive abilities before 36 weeks PMA compared to after (X2 = 4.500, *p* = 0.0339). Oxygen requirements at 28 days gestational age (X2 = 9.256, *p* = 0.0023), OR respiratory support at 36 weeks PMA (X2 = 9.256, *p* = 0.0023), NICHD 2001 (X2 = 18.35, *p* < 0.0001), Shennan (X2 = 4.130, *p* = 0.0421), and NICHD 2018 (X2 = 16.10, *p* < 0.0001) also had improved predictive ability after 40 weeks PMA. In general, patients of younger postnatal age and PMA at diagnosis fulfilled more definitions (Fig. 4). PMA was significantly different in the percentage of definitions fulfilled, specifically with PMA 27–30 weeks fulfilling significantly more definitions than PMA 42–45 weeks (one-way ANOVA, *p* = 0.0232, multiple comparisons test: *p* = 0.0238).

**Table 2:** Pairwise definition agreement

	Shennan	NICHD 2001	Isayama	NICHD 2018	Jensen	O2 at 28 days postnatal	resp support at 36wk PMA
Shennan	N/A	N/A	N/A	N/A	N/A	N/A	N/A
NICHD 2001	76.84%	N/A	N/A	N/A	N/A	N/A	N/A
Isayama	84.21%	69.47%	N/A	N/A	N/A	N/A	N/A
NICHD 2018	88.42%	77.89%	82.11%	N/A	N/A	N/A	N/A
Jensen	92.63%	76.84%	87.37%	89.47%	N/A	N/A	N/A
O2 at 28 days postnatal	89.47%	78.95%	86.32%	91.58%	92.63%	N/A	N/A
resp support at 36wk PMA	92.63%	76.84%	88.42%	92.63%	95.79%	96.84%	N/A
O2 OR resp support at 36wk PMA	89.47%	78.95%	86.32%	91.58%	92.63%	98.95%	96.84%

**Fig. 5:**
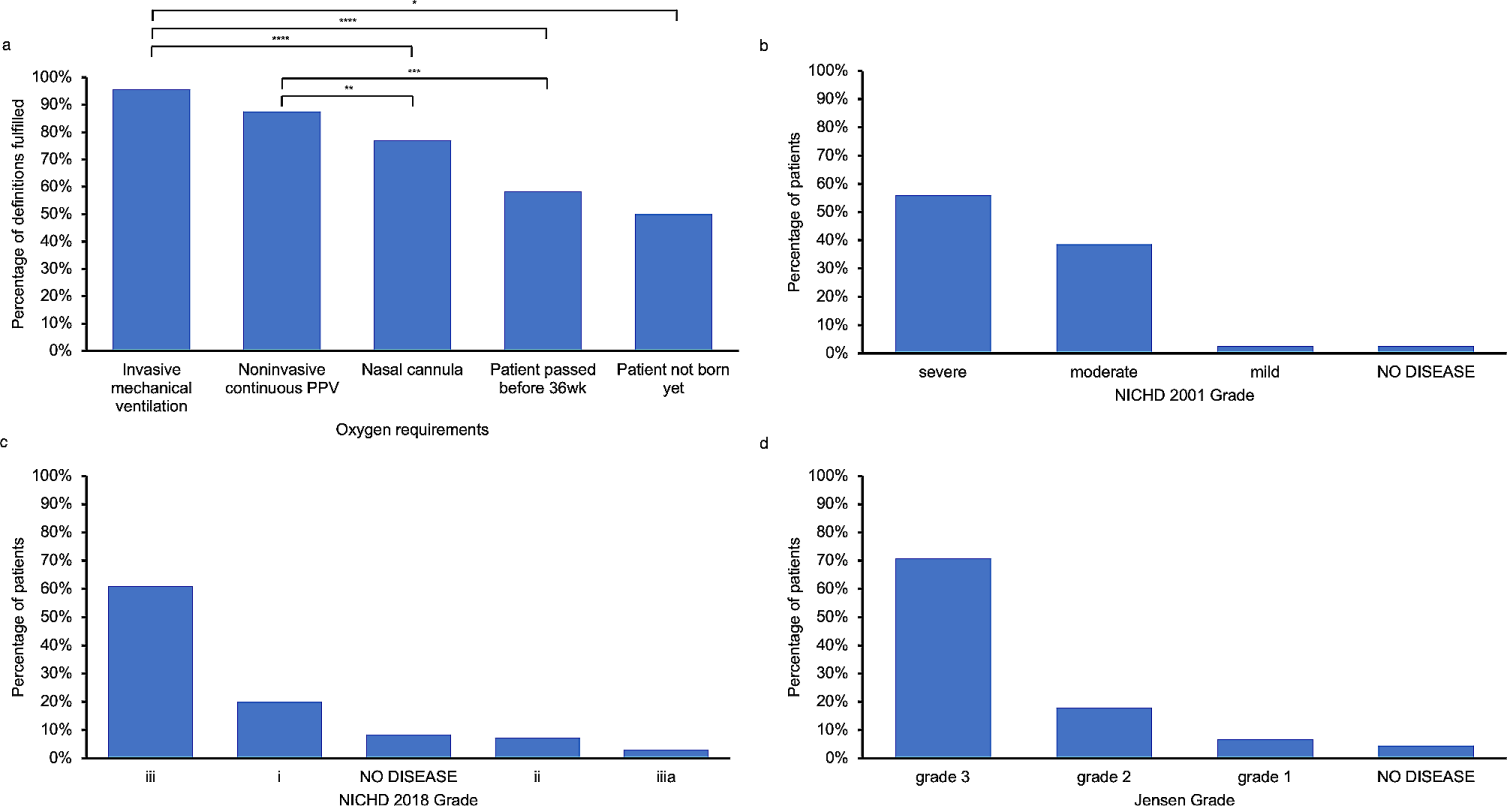
Graded definition severity distributions. a Diagnostic accuracy by oxygen requirements at 36 weeks postmenstrual age. b Grade distribution for NICHD 2001 definition. c Grade distribution for NICHD 2018 definition. d Grade distribution for Jensen definition

In addition to postnatal age and PMA, less severe oxygen (O2) requirements at 36 weeks PMA and bigger gestational age were correlated with poorer performance of the clinical definitions (one-way ANOVA, *p* < 0.0001, Fig. 5a). Individuals placed on nasal cannula O2 satisfied significantly fewer definitions compared to those on invasive mechanical ventilation (multiple comparisons test, *p* < 0.0001) and noninvasive continuous positive pressure ventilation (PPV) (multiple comparisons test, *p* = 0.0044). Correspondingly, for the definitions which involved a grading system based on severity (NICHD 2001, NICHD 2018, and Jensen), the patients in this study generally fit into more severe grades (Fig. 5b-d). 55% of patients fit into the severe grade for NICHD 2001, 78% fit into grade iii-iiia for NICHD 2018, and 70% fit into grade 3 for Jensen (Fig. 5b-d).

## Discussion

Some of the most important findings of our study include the high sensitivity of the definitions in capturing CLD cases as identified by radiologists, the preferencing of severe cases in radiologic diagnosis, and the ability to diagnose the condition early in the infant’s life. The low proportion of mild cases is reflected in the small number of patients on no oxygen or nasal cannula at 36 weeks PMA as opposed to invasive mechanical ventilation or noninvasive PPV despite the importance of nasal cannula settings in the diagnostic decision-making of most definitions. Given these data, it seems that typically the more severe cases of CLD are the ones that are distinctly noticeable on CXR, indicating high specificity of this imaging modality for diagnosing CLD. All clinical definitions fit the patients in this study very well, as most definitions consider PPV, invasive mechanical ventilation, or high-flow oxygen to be diagnostic of CLD. The nuance among definitions comes with defining the role of radiology in diagnosis, the time point at which to identify respiratory requirements, and the decision of whether to include early oxygen use as a criterion.

The role of chest x-ray in early of detection of chronic lung disease relies on the radiologist to recognize how the pathophysiology of the disease manifests on a chest x-ray. When premature infants require long standing intubation with high positive airway pressures, inevitably their alveoli distend and overtime the radiologist will see linear opacification of the interstitium as a result signifying thickening and scarring. If air dissects into the interstitium, the result will also be superimposed cystic lucencies that the radiologist can detect on the plain film. Interstitial opacities with or without cystic lucencies can signify chronic lung disease which the radiologist can detect once it manifests, helping the referring clinician use markers described in this paper to attach the diagnosis of chronic lung disease.

Due to the accuracy of radiology diagnosis of CLD, it seems that CXR analysis could be useful in determining a CLD diagnosis before some of the traditional time points are reached. The majority of cases were correctly diagnosed by radiologists before 36 weeks PMA despite not having any mention of CLD in the patient’s background, signaling that there is no need to wait until this time to make a definitive diagnosis in these patients. Patients diagnosed earlier actually had a positive correlation with better fit among the clinical definitions, although this could be due to the severity of these cases which allowed for less ambiguity, as opposed to more nuanced cases which did not surface on imaging until later in life. A combination of respiratory requirements and findings on CXR could potentially be used in determining a CLD diagnosis well before 36 weeks PMA.

Additionally, lung ultrasound is a technique recently applied to the diagnosis of CLD and provides a low-cost, low-risk method of diagnosing or predicting CLD as early as 7–14 days of life [24]. Some diagnostic hallmarks of CLD on lung ultrasound include a thickened pleural line, an interstitial-alveolar pattern, and consolidations [24]. However, currently lung ultrasound is not a specific technique, and is most powerful when combined with other clinical criteria, CXR, and predictors of respiratory outcome [24].

Of the CLD cases with equivocal diagnoses, symptomatology tended to be mild. As to whether or not these mild cases are correct in being identified as CLD or non-CLD cases should be determined by the ability of this label to predict long-term pulmonary outcomes. One study by Sun et al. analyzed the ability of each class of the 2001 and 2018 NICHD CLD definitions in predicting long-term pulmonary outcomes including supplemental O2 use, ventilator use, and ≥ 2 respiratory-related hospitalizations. This study found approximately 16% of mild cases as classified by NICHD 2001 and 20% of mild cases as classified by NICHD 2018 to demonstrate late death or a serious respiratory morbidity, with the vast majority being ≥ 2 respiratory-related hospitalizations [25]. Given that these percentages of adverse respiratory outcomes are significantly higher than the general public, the argument could be made that mild cases of CLD are important to correctly diagnose, which could justify the need to increase the sensitivity of CXR in detecting mild cases.

While we aimed to be comprehensive in the inclusion of CLD clinical definitions, we excluded the definitions involving physiologic tests by Walsh [15] and Svedenkrans [16] due to the lack of documented data of physiologic tests in our cohort. The Walsh test was shown to reduce CLD diagnosis rates compared to the NICHD 2001 definition, and the Svedenkrans test added further nuance to the grading system of the NICHD 2018 definition. Given the severe nature of respiratory disease in the patients in this study and the stratification by grading among three other definitions, it is unlikely that either definition would have added unique insight into data analysis. However, refined grading and precise diagnosis of borderline cases are generally essential for outcome prediction and disease management.

Strengths of this study include the wide selection of definitions compared, the comprehensive inclusion of eligible patients, and the unique focus on the radiologic diagnostic accuracy of CLD per these clinical definitions. Limitations include the smaller size of the patient population and the exclusion of patients undiagnosed with CLD by radiologists. Given that radiologists tend to preference more severe cases in their diagnoses, it could be interesting to conversely study patients undiagnosed with CLD by radiologists but with high-risk signs of disease otherwise (need for respiratory support, fitting into the CLD clinical definitions, previously diagnosed, presence of CLD-like terms in radiology report, etc.). While outcome prediction by radiology diagnosis of CLD can be mediated by precedent work done in correlating each definition with outcomes [10, 20–23, 26–29], further work can be done to directly correlate the association between radiology findings and patient outcomes. Additionally, more work can be done in categorizing associations between each definition and its predictive ability of morbidity and mortality, as well as in composing further refined definitions of CLD designed to encapsulate this stratification of outcomes.

## Conclusion

Radiologists tend to diagnose more severe cases of chronic lung disease of prematurity on chest x-ray, which are appropriately categorized under all commonly used clinical definitions. Given the accuracy of radiologic diagnoses so early in an infant’s development, there is justification for diagnosis to potentially take place sooner when there are signs of lung disease on chest x-ray in patients needing positive pressure or mechanical ventilation.

### Electronic supplementary material

Below is the link to the electronic supplementary material.

Supplementary Material 1

Supplementary Material 2

## Data Availability

All data generated or analysed during this study are included in this published article [and its supplementary information files].

## Abbreviations

ANOVA: Analysis of variance
CHLA: Children’s Hospital Los Angeles
CLD: Chronic lung disease of prematurity
CXR: Chest x-ray
NICHD: National Institute of Child Health and Human Development
O2: Oxygen
PMA: Postmenstrual age
PPV: Positive pressure ventilation
