# Supplementary figures and images for: Assessing the agreement of chronic lung disease of prematurity diagnosis between radiologists and clinical criteria
Source: Matern Health Neonatol Perinatol. 2024 Apr 5;10:8. doi: 10.1186/s40748-024-00178-4 (PMC10996264; doi:10.1186/s40748-024-00178-4)

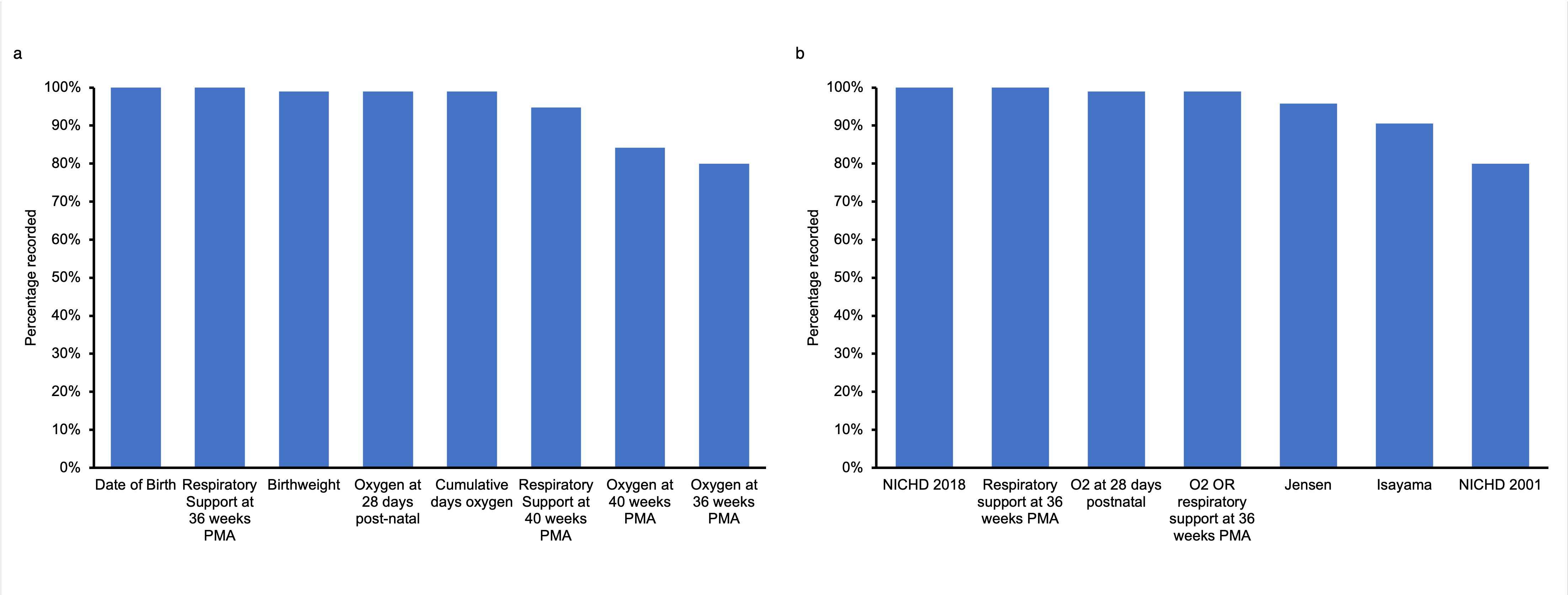

Supplement: Supplementary file 2 — Supplementary Material 2 [file 40748_2024_178_MOESM2_ESM.tiff]
